# Antenna selection for multiple-input multiple-output systems based on deep convolutional neural networks

**DOI:** 10.1371/journal.pone.0215672

**Published:** 2019-05-01

**Authors:** Jia-xin Cai, Ranxu Zhong, Yan Li

**Affiliations:** 1 School of Applied Mathematics, Xiamen University of Technology, Xiamen, P.R. China; 2 Department of software research and development, Guangdong Grandmark Automotive Systems CO., LTD, Dongguan, P.R. China; 3 School of Opto-electronic and Communication Engineering, Xiamen University of Technology, Xiamen, P.R. China; Newcastle University, UNITED KINGDOM

## Abstract

Antenna selection in Multiple-Input Multiple-Output (MIMO) systems has attracted increasing attention due to the challenge of keeping a balance between communication performance and computational complexity. Recently, deep learning based methods have achieved promising performance in many application fields. This paper proposed a deep learning (DL) based antenna selection technique. First, we generated the label of training antenna systems by maximizing the channel capacity. Then, we adopted the deep convolutional neural network (CNN) on the channel matrices to explicitly exploit the massive latent cues of attenuation coefficients. Finally, we used the adopted CNN to assign the class label and then select the optimal antenna subset. Experimental results demonstrate that our method can achieve better performance than the state-of-the-art baselines for data-driven based antenna selection.

## Introduction

Antenna system has been widely used in many application fields, such as public transportation, shopping malls, smart building, automotive radar, satellite communications, airplane landing, and astronomy [1–5]. The multiple-input multiple-output (MIMO) system also has received increasing interest in the area of wireless communication over the past few decades. Due to the rapid increasing of cellular mobile device usage and the limitation of computing power, antenna selection has attracted more and more attention recently. Antenna selection can keep a balance between communication performance and computational complexity. It can reduce the hardware cost and computational complexity, and keep enough gain rate or signal-to-noise ratio (SNR) at the same time. Usually, obtaining the optimal antenna subset needs to compare all possible combinations by exhaustive searching. It takes great amount of calculation and is very time-consuming. Because the exhaustive searching methods are impractical, many suboptimal models have been proposed. In general, existing methods can be categorized as two types, optimization-driven methods and data-driven methods.

The optimization-driven methods use the communication quality criteria, such as bit-error-rate (BER), channel capacity or bit error ratio, as the objective of antenna subset selection [6–10]. The optimization problems of antenna selection are always non-convex. Convex relaxation is one solution for sub-optimization problem. Because the results of convex relaxation depend on the initial points, convex relaxation based methods easily fall into local optimum [11]. Furthermore, the convex relaxation algorithms are still of high computational complexity. They are not suitable for real-time antenna selection. Some intelligent searching algorithms, such as simulated annealing algorithm [12], genetic algorithm [13] and particle swarm optimization algorithm [14], are also employed to find a suboptimal solution. The intelligent searching algorithms avoid the problem of falling into local optimum. But these algorithms cost much more time than local optimization algorithms. Overall, optimization-driven methods usually meet limitations in real-life application.

Data-driven methods use the data mining techniques to obtain the best antenna subset [15]. These methods apply supervised machine learning on big data. Given sufficient channel data, classifiers such as k-nearest neighbors (KNN) and support vector machine (SVM), can be trained to map the channel data into the selected antenna subset. Joung [15] has proven that the communication performances of KNN and SVM exceed that of conventional optimization-driven methods. Compared to optimization-driven methods, data-driven models have smaller computational cost in the test stage. Data-driven methods are more convenient to be implemented in real-life application.

Recently, deep learning (DL) techniques have received strong interest due to their promising performance for large-scale data analysis [16–19]. Deep artificial neural networks (DNNs) [16] are more powerful than traditional three-layer artificial neural networks (ANNs) (such as perceptron and Hopfield network). DNNs are more capable to reduce the massive variation of input signals. This property helps the DNNs win tremendous contests in the field of data analysis. Convolutional Neural Network (CNN) [17], a classical feed-forward ANN, is usually used to build deep network architectures. The most popular architectures of CNN are LeNet [20], AlexNet [17], VGG-16 [21] and ResNet [22]. CNN has resulted in significant progress in many large-scale data analysis tasks such as image classification, video analysis, handwritten digit recognition [23], face recognition [24], action recognition [25], recommender systems [26], natural language processing [27], and other data-driven tasks. There are existing works employing DL for wireless communication [28–32]. Application of DL to antenna selection, however, has no readily available experience.

Maximizing channel capacity is a common criterion for receiving antenna selection. Channel matrix is often used to represent the channel capacity of MIMO system. Channel matrix is a highly structured and high-dimensional data with a lot of texture information and hidden details. In the filed of image processing, image data also have high dimensions and lots of texture information. This fact inspired us to adopt channel matrix by CNN. This work proposed a data-driven receiving antenna selection approach through deep CNN and channel capacity criterion. As an extension of our previous conference report [33], this work attempted to introduce the DL techniques into the field of antenna selection. We adopted deep CNN on channel matrix to extract rich features for antenna selection. The trained deep CNN can easily assign the best antenna subset selection solution to test samples. The procedure of our model includes three steps. Firstly, the labels of channel matrices are generated by maximizing the channel capacity for all training samples. Secondly, a deep CNN that explicitly exploits the massive latent cues of attenuation coefficients is learned. Finally, the adopted deep CNN is used to classify the test channel matrix and select the optimal antenna subset. Experimental results show the effectiveness of deep CNN for antenna selection. The deep CNN outperforms baseline methods for both the classification performance and communication performance.

The contributions of our work are listed as follows.

1. We introduced the DL techniques into the field of wireless antenna selection. An antenna section model based on deep CNN and channel capacity criterion was proposed.
2. We adopted an LeNet CNN and a ResNet CNN on the training channel matrices respectively. The deep CNNs were trained on massive training antenna systems. The output of deep CNNs were converted to the solution of antenna selection for test channel matrices.
3. The proposed approaches have better classification performance and communication performance than state-of-the-art methods. The proposed models can be implemented on real-life applications in future and have potential applications.

The rest of this paper is organized as follows. In Section 2, we discuss some related work. In Section 3, we describe the system model. In Section 4, we describe the labeling system and the network architecture. Section 5 gives the details of experimental setup and the analysis. Finally, in Section 6 our final remarks are presented.

## Relative works

### Antenna selection

#### Optimization-driven methods

Optimization-driven methods employ suboptimal search algorithms to find the best subset. Padmanabhan et al. [6] considered the problem of receive antenna selection using the known temporal correlation of the channel symbols embedded in the data packets; the model was stated as a problem of minimizing the average packet error rate and converted to a partially observable Markov decision process framework which was solved by heuristic searching schemes. Gulati and Dandekar [7] stated the antenna state selection problem as a multi-armed bandit problem, and used the criterion of optimizing the arbitrary link quality metrics to solve it. Zhou et al. [8] introduced simple near-optimal Min-Max criterion and selected antenna combinations based on maximum sum-rate (Max-SR) and minimum symbol-errorrate (Min-SER) criterions. Yan et al. [9] modeled the trade-off between feedback overhead and secrecy performance by maximizing SNR of the transmitter-receiver channel.

#### Data-driven methods

Another way is the data-driven methods which employ supervised machine learning algorithms. Joung [15] used KNN and SVM for antenna selection in wireless communication. KNN and SVM were compared to two conventional optimization-driven methods, Max-min eigenvalue and Max-min channel norm. The experiments have shown that the communications performance of KNN and SVM exceed that of Max-min eigenvalue and Max-min channel norm. Additionally, the computational complexity of KNN and SVM is much lower than that of Max-min eigenvalue and Max-min channel norm.

### Deep learning

Recently, DNNs have been employed in many application fields. It has been proved that deep network architectures have amazing processing power even superior to human. DNN [16] with multiple middle layers, has achieved great success on a wide variety of multi-media classification such as image recognition, video classification, speech recognition and natural language processing. CNN [17], an alternative type of DNN, is a more effective network. CNN can capture spatial and temporal correlation of data with little parameters. Recurrent Neural Network (RNN) [18], and its effective variant, Long Short-Term Memory Network (LSTM) [19], are also popular in language modeling.

## System model

Consider an MIMO system with *N*_*t*_ transmit antennas and *N*_*r*_ receive antennas. The channel matrix is denoted as $H=\left[h_{ij}\right]\in C^{N_{r}*N_{t}}$, where *h*_*ij*_ is the attenuation coefficient between the *j*th transmit antenna and the *i*th receive antenna. Let $r\left(k\right)={\left[r_{1}\left(k\right),r_{2}\left(k\right),\ldots ,r_{N_{r}}\left(k\right)\right]}^{T}$ denote the received signal, $t\left(k\right)={\left[t_{1}\left(k\right),t_{2}\left(k\right),\ldots ,t_{N_{t}}\left(k\right)\right]}^{T}$ denote the transmitter signal, and $w\left(k\right)={\left[w_{1}\left(k\right),w_{2}\left(k\right),\ldots ,w_{N_{r}}\left(k\right)\right]}^{T}$ denote the white Gaussian noise. Here *k* denotes the time count of a discrete time signal. Denote the SNR as *ρ*, then the MIMO system model can be presented as follows.

$$\begin{array}{c} \begin{array}{c} r\left(k\right)=\sqrt{\frac{\rho }{N_{t}}}H*t\left(k\right)+w\left(k\right) \end{array} \end{array}$$ (1)

Assuming the channel matrix is known at the receiving terminal, and unknown at the transmitting terminal. The aim is to select *N*_*s*_ receiving antennas from the *N*_*r*_ receiving antennas so that the channel capacity is maximal. Let $B\in C^{N_{s}*N_{t}}$ denote the partial channel matrix whose rows are selected from original channel matrix *H*. The objective function can be written as follows.

$$\begin{array}{c} \begin{array}{c} max_{B}C\left(B\right)=log_{2}det\left(I_{N_{t}}+\frac{\rho }{N_{t}}B^{H}B\right) \end{array} \end{array}$$ (2)

where *C*(*B*) denotes the channel capacity of *B*, and $I_{N_{t}}$ is the identity matrix of size *N*_*t*_. *B*^*H*^ denotes the Hermitian conjugate of *B*.

## Learning deep CNN for antenna selection

### Label generation

#### Data normalization

A channel matrix is seen as a data sample. Firstly, the complex channel matrix *H* is converted to a real-value matrix by substituting the (*i*, *j*)th element *h*_*ij*_ with its amplitude |*h*_*ij*_|.

$$\begin{array}{c} \begin{array}{c} h_{ij}⟵\left|h_{ij}\right|,\;\;\;for\;all\;i,j \end{array} \end{array}$$ (3)

Because each row of *H* denotes a receiving antennas, we need to perform normalization for each row vector. So the channel matrix is normalized to obtain a scale invariant feature as follows.

$$\begin{array}{c} \begin{array}{c} h_{ij}⟵\frac{h_{ij}-\min\left\{h_{i}\right\}}{\max\left\{h_{i}\right\}-\min\left\{h_{i}\right\}},\;\;\;for\;all\;i,j \end{array} \end{array}$$ (4)

Here *h*_*i*_ denotes the *i*-th row vector of *H*.

#### Channel matrix labeling for training samples

Suppose there are *W* ways to select *N*_*s*_ rows from *N*_*r*_ rows. Denote every combination of the way to select *N*_*s*_ receiving antennas from *N*_*r*_ receive antennas as a pattern class. Each combination is mapped to a pattern class. Then the total number of class labels is $W=C_{N_{t}}^{N_{r}}$.

Some examples of one-to-one match between selected antenna indices and class labels are shown in Table 1. If the selected antenna index is (1, 2, …, *N*_*s*_ − 1, *N*_*s*_), then the corresponding class can be defined as 1. If the selected antenna index changes to (1, 2, …, *N*_*s*_ − 1, *N*_*s*_ + 1), then the corresponding class can be defined as 2. Repeat this process until all possible selected antenna indices have been gone through. Then every antenna selection scheme corresponds with one pattern label.

**Table 1:** Examples of selected antenna indices and their corresponding classes.

Selected antenna index	Corresponding class
(1, 2, …, *N*_*s*_ − 1, *N*_*s*_)	1
(1, 2, …, *N*_*s*_ − 1, *N*_*s*_+ 1)	2
…	…
(1, 2, …, *N*_*s*_ − 1, *N*_*r*_)	r-s+1
(1, 2, …, *N*_*s*_ − 2, *N*_*s*_, *N*_*s*_+1)	r-s+2
(1, 2, …, *N*_*s*_ − 2, *N*_*s*_, *N*_*s*_+2)	r-s+3
…	…

The goal is to find a combination *c*_*i*_ ∈ {*c*_1_, *c*_2_, …, *c*_*W*_} to select the rows {*H*_*c*_*i*_(1)_, *H*_*c*_*i*_(2)_, …, *H*_*c*_*i*_(*N*_*s*_)_} from the channel matrix *H* and build a new matrix $B_{c_{i}}={\left[H_{c_{i}\left(1\right)},H_{c_{i}\left(2\right)},\ldots ,H_{c_{i}\left(N_{s}\right)}\right]}^{T}$. For a channel matrix *H* in the training set, its class label *y*(*H*) is determined by using the following formula.

$$\begin{array}{c} \begin{array}{c} y\left(H\right)=argmax_{c_{i}\in \left\{c_{1},c_{2},\ldots ,c_{W}\right\}}C\left(B_{c_{i}}\right) \end{array} \end{array}$$ (5)

**Fig 1:**
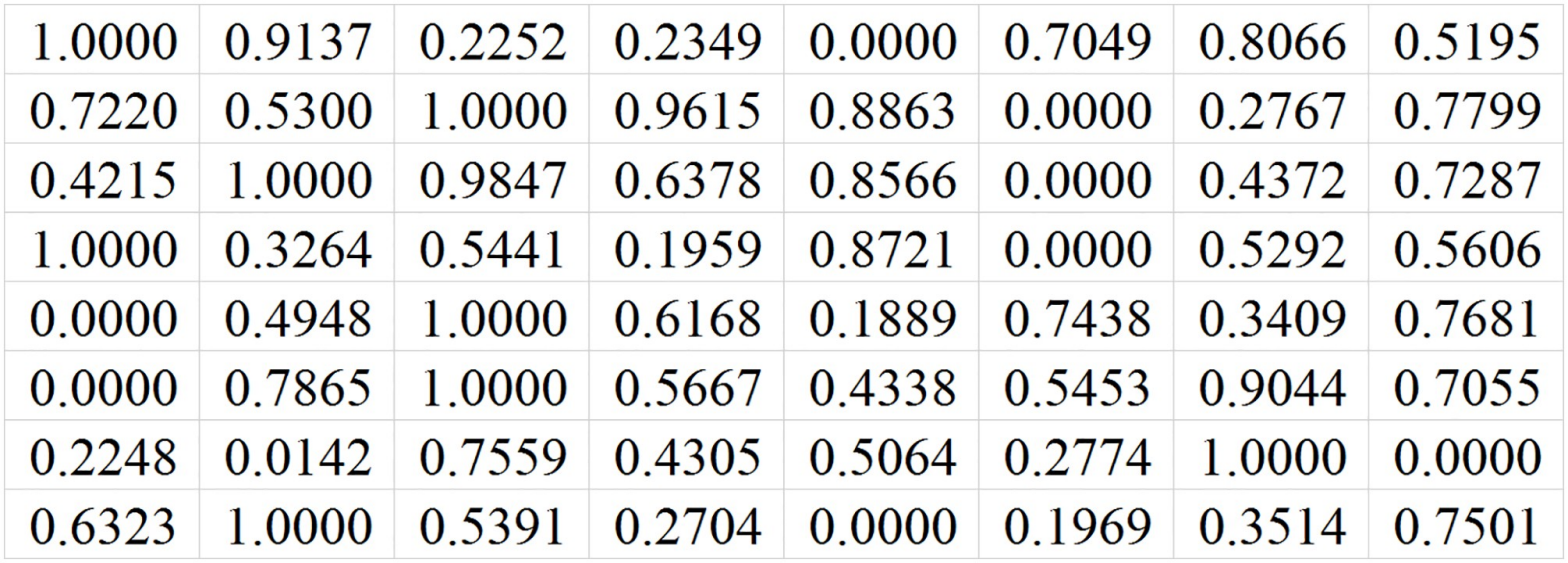
An example of channel matrix sample whose label is 1.

**Fig 2:**
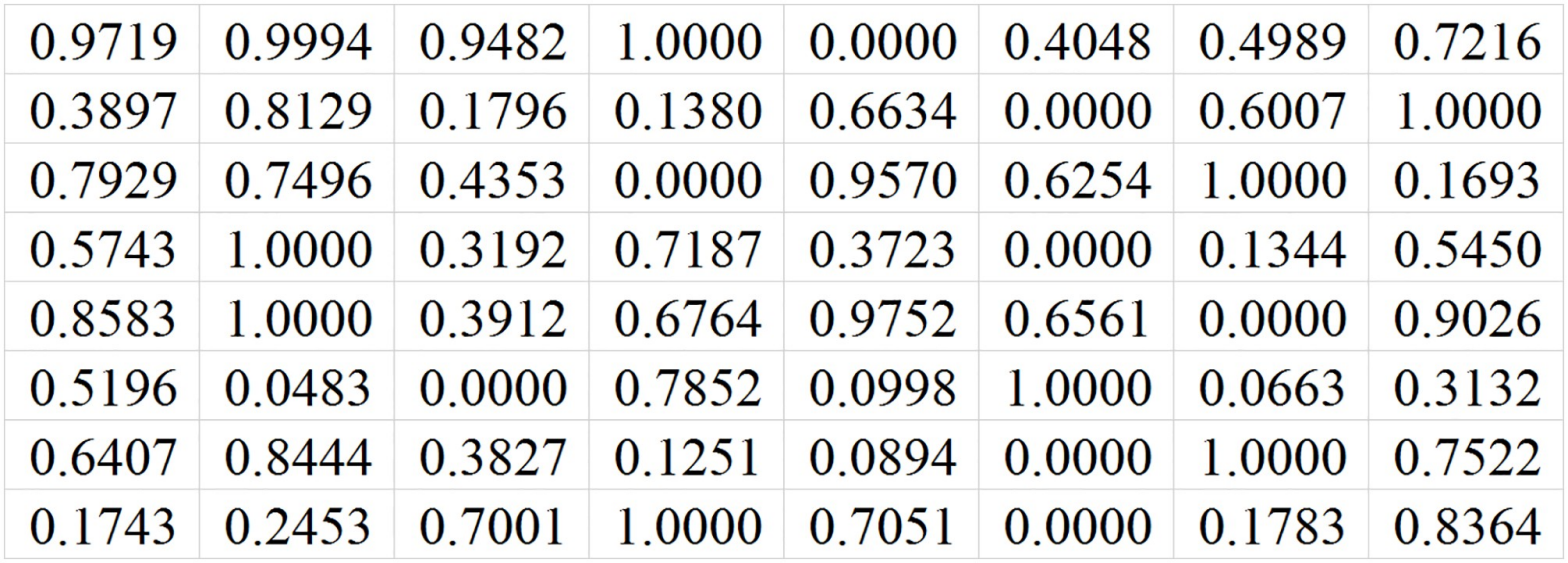
An example of channel matrix sample whose label is 2.

Fig 1 shows an example of channel matrix sample. *N*_*t*_ is 8, *N*_*r*_ is 8, and *N*_*s*_ is 2. The label is 1. That means the best antenna selection scheme is to select the first antenna and the second antenna. Fig 2 shows another channel matrix sample. *N*_*t*_ is 8, *N*_*r*_ is 8, *N*_*s*_ is 2, and the label is 2. The best antenna selection scheme is to select the first antenna and the third antenna.

### Deep CNN architecture

#### LeNet architecture

**Fig 3:**
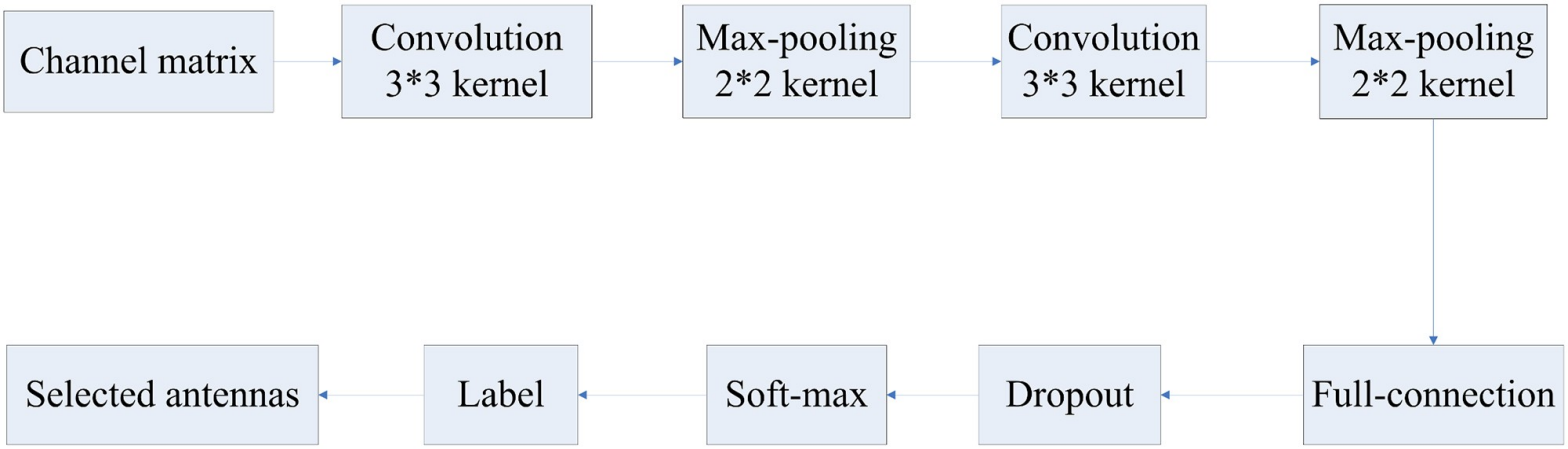
The framework of LeNet based antenna selection.

We employed a LeNet architecture CNN to adopt channel matrices. The framework of LeNet based antenna selection is illustrated in Fig 3. The communication parameters {*N*_*t*_, *N*_*r*_, *N*_*s*_} in the evaluation experiments were set as {8, 8, 2}. There are seven layers contained in LeNet, including two convolutional network layers, two pooling layers, a fully-connected network layer, a dropout layer, and a soft-max layer. The input of the convolutional neural network is an 8 × 8 channel matrix *H*. The first convolutional layer filters the 8 × 8 input channel matrix with 32 kernels of size 3 × 3. Then the first pooling layer is introduced to take the response of the first convolutional layer as input. It normalizes and pools the input into a 3 × 3 × 32 output response. The max-pooling kernels have a size of 2 × 2 and a stride of 2. The second convolutional layer filters the input response with 64 kernels of size 3 × 3. Then the second pooling layer converts the input response to a 2 × 2 × 64 output response. The max-pooling kernels have a size of 2 × 2 and a stride of 2. The fifth layer is the full-connected layer, which is a dense layer accelerating the convergence. It has 1024 full-connected kernels of size 1 × 1. Then a dropout layer, which randomly reset the output of each hidden neuron to zero with probability 0.5, is added behind the full-connected layer. The dropout layer prevents hidden units from relying on specific inputs and solves the over-fitting problem by employing ensemble learning technology. The response of dropout layer is fed to a soft-max layer, which produces a class label. There is an one-to-one correspondence between a class label and an antenna selection result. So we can use the output class label to find the solution of antenna section.

The Rectified Linear Unit(ReLU), which form is *f*(*x*) = *max*(0, *x*), was employed as the nonlinear activation function of all convolutional layers and the fully-connected layer. The cross entropy was employed as the loss function. The gradient descent optimizer was employed. The batch size was set as 10. The learning rate was set as 0.001. The training loop number was set as 1000000.

#### ResNet architecture

We have also employed a ResNet architecture for antenna selection. The input is an 8 × 8 input channel matrix. It is connected by a convolutional layer which has 64 kernels of size 3 × 3. Then a bottleneck convolutional layer is connected. The number of layer blocks is 3; the number of convolutional filter of the bottleneck convolutional layer is 16; the number of convolutional filters of the layers surrounding the bottleneck layer is 64. After that, the second bottleneck convolutional layer is connected. The number of layer blocks is 1; the number of convolutional filter of the bottleneck convolutional layer is 32. the number of convolutional filters of the layers surrounding the bottleneck layer is 128. Then the third bottleneck convolutional layer is connected. The number of layer blocks is 2; the number of convolutional filter of the bottleneck convolutional layer is 32; the number of convolutional filters of the layers surrounding the bottleneck layer is 128. After that, the fourth bottleneck convolutional layer is connected. The number of layer blocks is 1; the number of convolutional filter of the bottleneck convolutional layer is 64; The number of convolutional filters of the layers surrounding the bottleneck layer is 256. Then the fifth bottleneck convolutional layer is connected. The number of layer blocks is 2; the number of convolutional filter of the bottleneck convolutional layer is 64; The number of convolutional filters of the layers surrounding the bottleneck layer is 256. The response is then taken into an global average pooling layer. Then a fully connected layer is connected.

The ReLU unit was employed as the activation function of all convolutional layers. The soft-max function was employed as the activation function of fully connected layer. The cross entropy was employed as the loss function. The momentum optimizer was employed to train the network. The learning rate was set as 0.1. The batch size was set as 1280. The training loop number was set as 20.

### Implementation

The proposed method was coded in Python on a Windows 7 SP1 OS. CNNs were implemented using the TensorFlow framework. The experiments were performed on a computer with CPU Intel Xeon E5-2660 @ 2.2 GHz, GPU NVIDIA GTX1080Ti, and 64 GB of RAM.

## Experimental results and analysis

### Experimental setup

#### Simulated data

To evaluate the performance of the proposed method, we randomly generated 500000 channel matrix samples by i. i. d. sampling from a complex Gaussian distribution with mean 0 and variance 1. The number of transmit antennas *N*_*t*_ was set as 8. And the number of receive antennas *N*_*r*_ was set as 8 too. The number of selected receive antennas *N*_*s*_ was set as 2. The SNR was set as 10 dB. The heuristic searching method was used to generate the true class labels of all channel matrix samples. The data are public and can be download at https://pan.baidu.com/s/1O8G29t6IcvwfChr-DAQ1Cw.

#### Compared methods

KNN and SVM were employed as the compared methods. In the KNN classifying experiment, Euclidian distance was used as the metric measurement. KNN classifier labels the query sample by assigning the most common class among its *K* nearest neighbors to it. The parameter *K* was tuned by exhaustive searching. The classification results on the test set were recorded to obtain optimal solutions of antennas selection. For SVM, the radial basis function (RBF) was employed as the kernel function; the parameter gamma in the RBF kernel function and the cost parameter were tuned by grid searching; the one-vs-rest strategy was employed to extend the binary-class classification problem to the multi-class classification problem; the classification result of SVM was recorded to get the optimal antennas subset.

RNN and LSTM were also employed as the compared methods. For RNN, the learning rate was set as 0.001, the training loop number was set as 500000, the batch size was set as 100, the time-step was set as 8, and the number of hidden states was set as 100. For LSTM, the learning rate was set as 0.01, the training loop number was set as 500, the batch size was set as 100, the time-step was set as 8, and the number of hidden states was set as 100.

Other CNN architectures, AlexNet and VGG-16, were also employed as the compared methods. For AlexNet, the learning rate was set as 0.001, the training loop number was set as 20, the batch size was set as 1280, and the “momentum” optimizer is employed. For VGG-16, the learning rate was set as 0.001, the training loop number was set as 20, the batch size was set as 1280, and the “rmsprop” optimizer is employed.

#### Evaluation

For LeNet, KNN and SVM, the five-fold cross-validation strategy was employed to tune the parameters and compute the evaluation results. The original data were randomly divided into five equal-sized groups. A single group was chosen as the test set, and the remaining four groups were employed as the training set. The cross-validation process was repeated five times so that each of the five subsample groups was employed exactly once as the test set. The classification accuracy was computed from the folds. Then the accuracies of all test folds were averaged to produce an overall accuracy, which provided an evaluation measure of different classifiers.

For VGG-16, AlexNet and ResNet, 1800000 samples were assigned to the test set, and the remain samples were assigned to the training set. For LSTM and RNN, 2000000 samples were assigned to the test set, and the remain samples were assigned to the training set. We computed the accuracy on the test set to evaluate the classifiers.

Moreover, we computed the loss of channel capacity after antenna selection. The channel capacity loss can be employed to evaluate the communication performance. For a test channel matrix *H*, the partial channel matrix $\hat{B}$ was generated according to the classification result. Then the channel capacity loss of *H* can be computed as follows.

$$\begin{array}{c} \begin{array}{c} L\left(H\right)=||C\left(H\right)-C\left(\hat{B}\right)\left|\right| \end{array} \end{array}$$ (6)

We computed the channel capacity loss of all test channel matrix samples, and then calculated the average channel capacity loss of the test set. The average channel capacity loss was employed as the criteria to evaluate the communication performance. Due to the computation of capacity loss cost much more time than that of accuracy, we use the accuracy to tune the parameters of CNN models, such as training loop number and batch size.

### Results and analysis

Table 2 shows the classification accuracies of CNNs and other compared methods. As seen in Table 2, ResNet provides an accuracy of 79.16%, and LeNet provides an accuracy of 49.21%. ResNet has an appealing accuracy and outperforms other listed methods. ResNet can effectively train a very deep network by residual learning. The increase of depth can bring a promising accuracy. The accuracies of AlexNet and VGG-16 are very low. AlexNet and VGG-16 architectures work well for image classification. However, it seems that they do not suit antenna selection data. The accuracies of RNN and LSTM are 24.00% and 60.00% respectively. The results imply that the antenna selection data can be treat as sequence data and are suitable for sequence model. The accuracy of RNN is low, but LSTM greatly increases the performance. That is because LSTM can remember the long-time information and solve the “vanishing gradient” problem. The accuracies of KNN and SVM are 8.29% and 22.12% respectively. Because the size of dataset is very large and the problem is highly non-linear, it is very hard for simple classifiers such as KNN and SVM to achieve a good accuracy.

**Table 2:** Comparison of antenna selection methods about classification accuracy.

Antenna selection method	Accuracy(%)
ResNet	79.16
LeNet	49.21
AlexNet	3.60
VGG-16	3.65
RNN	24.00
LSTM	60.00
KNN	8.29
SVM	22.12

Capacity performance are more important than accuracy. We compared the channel capacity loss of CNN methods to that of compared methods. The comparison results are showed in Table 3. As seen in Table 3, the average channel capacity loss of ResNet is 6.24 with variance 0.13; the average channel capacity loss of LeNet is 3.63 with variance 0.49; the average channel capacity loss of AlexNet is 6.76 with variance 0.18; the average channel capacity loss of VGG-16 is 6.78 with variance 0.16; the average channel capacity loss of RNN is 6.72 with variance 0.27; the average channel capacity loss of LSTM is 6.78 with variance 0.27; the average channel capacity loss of KNN is 7.08 with variance 0.47; the average channel capacity loss of SVM is 6.95 with variance 0.34. LeNet do not has the optimal accuracy, but it has the minimal channel capacity loss. Although ResNet has the best classification performance, it do not have much advantage in communication performance. Specially, AlexNet and VGG-16 have very low accuracies but real good communication performance. It shows that AlexNet and VGG-16 try to get sub-optimal labels for test samples in most cases. However, these sub-optimal labels correspond to an enough low channel capacity loss. Overall, the comparison results confirm that LeNet is better than others for antenna selection task. The results prove that using LeNet for antenna selection is a competitive and acceptable choice.

**Table 3:** Comparison of antenna selection methods about channel capacity loss.

Antenna selection method	Capacity loss	Variance
ResNet	6.24	0.13
LeNet	3.63	0.49
AlexNet	6.76	0.18
VGG-16	6.78	0.16
RNN	6.72	0.27
LSTM	6.78	0.27
KNN	7.08	0.47
SVM	6.95	0.34

For machine learning based antenna selection, the most difficult thing is that the map between channel matrix and the best antenna index is highly non-linear. Another difficulty is that the similarities between different samples are very small. So it is very hard for pattern classifiers to separate samples of different classes and get high accuracy. CNN provides an efficient way to mine the deep latent meanings of channel matrices. The strength of association between channel matrix and the best antenna index can be enhanced in the deep network architectures. So the deep representation will help the classifiers to approximate the complex map from channel matrix to the best antenna index.

The accuracy of LeNet is 49.21%, which seems to be low. However, the real purpose of antenna selection is to achieve the best communication performance instead of classification performance. For a test channel matrix, the true label means the optimal antenna selection scheme. However, for wireless communication, a suboptimal antenna selection scheme is still acceptable if the channel capacity loss is low. ResNet has the minimal misclassified samples. But ResNet has higher channel capacity loss on the misclassified samples than LeNet. LeNet is a less accurate approach, but enable the samples which are difficult to be classify to get a good-enough suboptimal label. Compared to other listed methods, LeNet has better communication performance and is more robust. In this sense, LeNet is better than ResNet and other compared methods for antenna selection task.

We have also analyzed the trade-off between accuracy and speed. Firstly, we have analyzed the relation between classification accuracy and training loop number of CNN (LeNet). The results are listed in Table 4. As shown in Table 4, the accuracy of CNN achieves 42.05% when the training loop number is set as 100000. If the training loop number is set as 500000, then the accuracy arises to 48.68%. That is a great improvement. However, the accuracy only slightly rises to 49.21% when the training loop number arises to 1000000. Increasing the number of training loops will cost more computation power in the training stage. However, the computation speed of real antenna selection system mainly depends on the test stage. And increasing the number of training loops will not lead to the computational cost increase of the test stage. The experimental results indicate that setting the training loop number as 500000 is an acceptable option. It can produce enough antenna selection performance and will not increased unnecessary time cost.

**Table 4:** Relation between of classification accuracy of antenna selection and number of training loops.

Training loop number	Accuracy(%)
100000	42.05
500000	48.68
1000000	49.21

Secondly, we have also analyzed the relation between accuracy and number of samples. For one dataset, 50000 samples are randomly sampled from a complex Gaussian distribution with mean 0 and variance 1, and the five-fold cross-validation strategy is employed to set the test sample size as 20% of the whole dataset. For another dataset, 2000000 samples are randomly sampled from a complex Gaussian distribution with mean 0 and variance 1, and the test sample size is set as 5% of the whole dataset. The comparison results are showed in Table 5. Experimental results show that large training sample size will lead to better antenna selection performance. However, a dataset of 500000 samples is sufficient for building a CNN based antenna selection system.

**Table 5:** Relation between CNN accuracy and number of samples.

Sample size	Test sample proportion(%)	Accuracy(%)
500000	20	49.21
2000000	5	49.98

We have also analyzed the training time and test time of CNN, RNN and LSTM. The training time on the whole training set are listed in Table 6. The training time of ResNet, AlexNet, VGG-16, RNN, LSTM are 5000 s, 4480 s, 1120 s, 6609 s and 7396 s respectively. VGG-16 has obvious advantages in training time. The average test time on one sample is also listed in Table 6. The test time of ResNet, AlexNet, VGG-16, RNN, LSTM are 0.015 s, 0.012 s, 0.012 s, 0.014 s and 0.015 s respectively. The test time required by these approaches are very close. It costs very little time to test one sample. Experimental results show that DL based antenna selection is real-time. The time to train LeNet model in one fold for five-fold cross-validation is about several hours in the current experimental setting. About 1 day are needed to implement the five-fold cross-validation. The training stage is hard to perform real-timely and need to be executed in the earlier step. It is a long way to realize a real-time CNN training system for antenna section. However, using a trained CNN to test a sample requires less than one second and can be executed real-timely.

**Table 6:** Comparison of antenna selection methods about training time and test time.

Antenna selection method	training time (s)	test time (s)
ResNet	5000	0.015
AlexNet	4480	0.012
VGG-16	1120	0.012
RNN	6609	0.014
LSTM	7396	0.015

**Fig 4:**
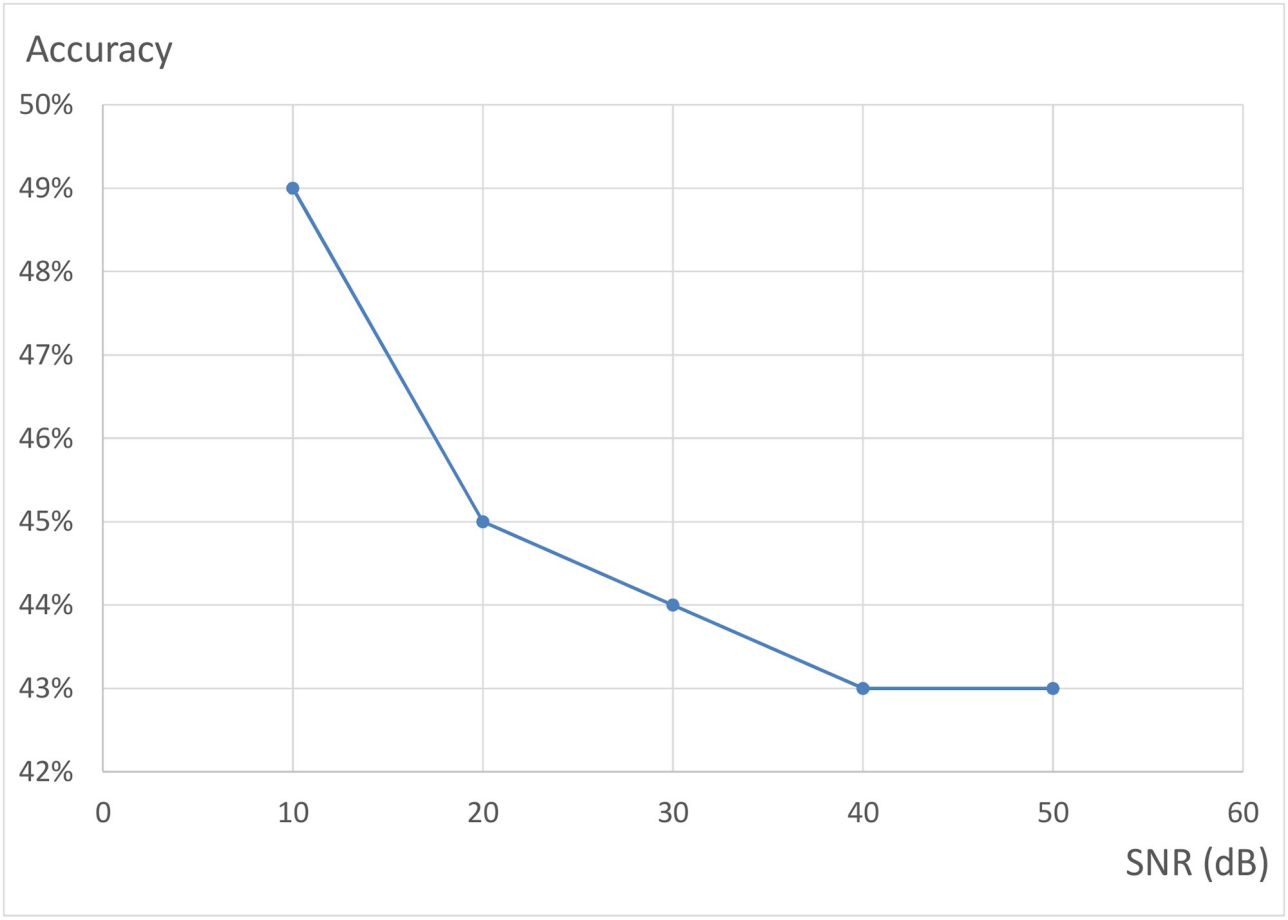
The relation between CNN accuracy and SNR.

**Fig 5:**
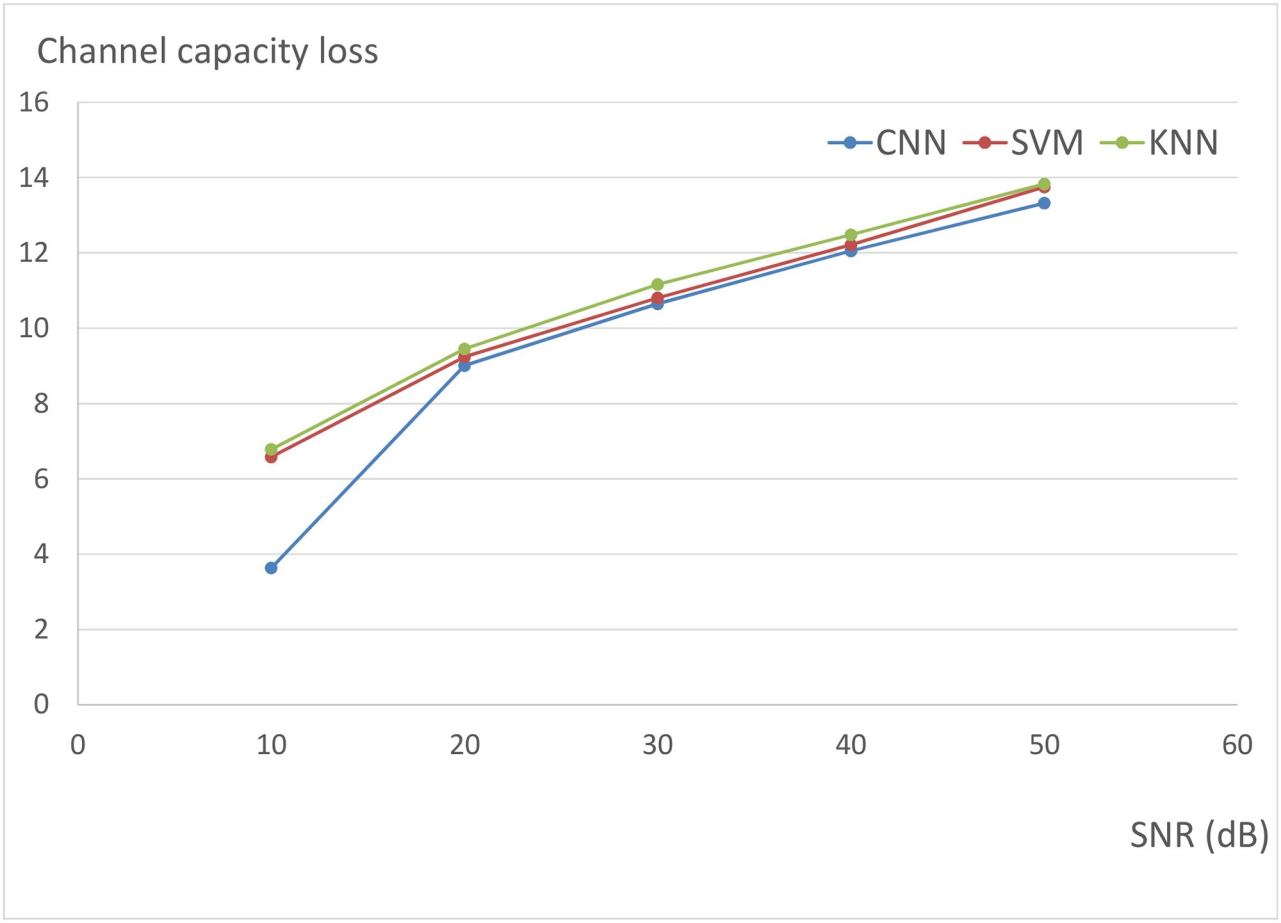
The relation between channel capacity loss and SNR.

**Fig 6:**
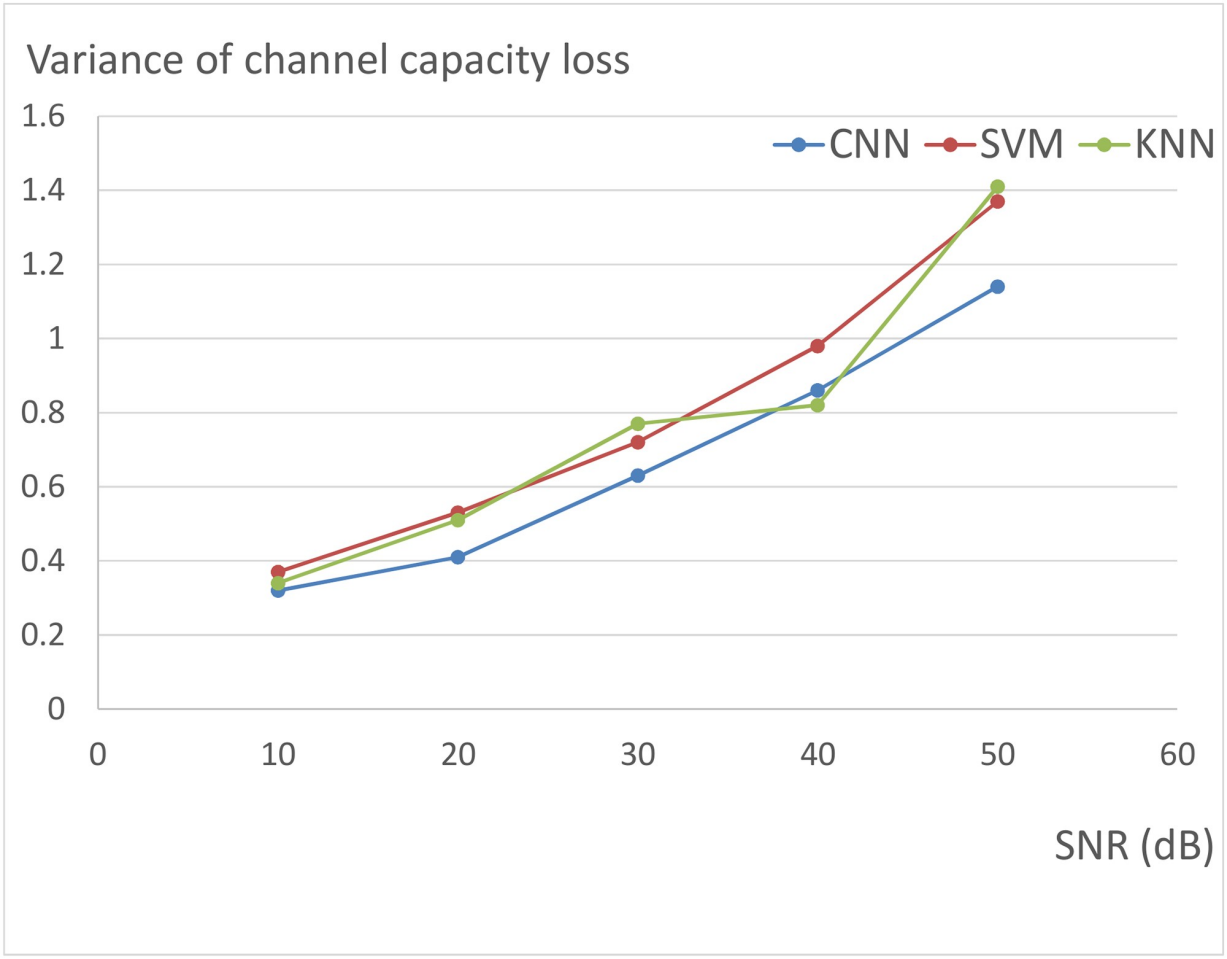
The relation between variance of channel capacity loss and SNR.

We have also analyzed the relation between SNR and the performance of CNN (LeNet architecture). Fig 4 shows the relation between CNN accuracy and SNR. Fig 5 shows the relation between channel capacity loss and SNR. And Fig 6 shows the relation between variance of channel capacity loss and SNR. If SNR rises from 10 dB to 50 dB, the accuracy and channel capacity loss will descend, and the variance of channel capacity loss will increase. However, the channel capacity loss of CNN is always less than that of SVM and KNN under the same condition. And the channel capacity loss variance of CNN is less than that of KNN and SVM in most cases. Experimental results demonstrated that CNN outperforms KNN and SVM for antenna selection.

This work is a preliminary work for validating the idea of selecting antennas in MIMO system by DL. The scale of test antenna system in our experiments is not large. If the number of transmit antennas and receive antennas increases, then the dimension of channel matrix will increase fast. It means that we should generated a larger dataset for training CNN. However, it requires a more expensive GPU with larger memory or even a GPU cluster. Our laboratory cannot afford the cost of a stronger computing hardware, so we only set the number of receive antennas to 8 and the selected receive antenna number to 2. However, the application of CNN based large scale antenna selection still has good future to expect.

Our work focus on data-driven antenna selection. So we only compared our method to state-of-the-art data-driven methods. We have not compared the proposed methods to optimization-driven methods. Detailed comparison between data-driven methods and optimization-driven methods has been presented in [15]. Joung [15] has proved that KNN and SVM can achieve reasonable performance for antenna selection in wireless communication. And our experiments have shown that the capacity performance of our method exceed that of KNN and SVM. Obvious, the computational complexity of CNN is much larger than KNN and SVM. Although we have not performed experiments on large scale antenna system, it can be convinced by the experience of digit image processing that the test time of CNN would be acceptable and much lower than that of optimization-driven methods on a large scale antenna system.

Another limitation of this work is that we used the simulated antenna data instead of real antenna data to test the deep learning based selection algorithms. However, real antenna data are expensive to obtain, and a rich supply of real antenna data is inaccessible for researchers. This is a common problem in the field of antenna selection. Most works in the field of antenna selection used the simulated data to test the effectiveness of antenna selection algorithms.

## Conclusion

This work introduced a receiving antenna selection framework based on deep CNNs and the channel capacity criterion. The proposed methods used convolutional structure to extract rich features from the channel matrices. CNNs were used to train powerful classifiers for selecting antennas. The proposed approach was validated on simulated antenna system data. The proposed method outperformed the state-of-the-art baselines. Our future work will include the improvement of deep networks and the evaluation on real-life antenna systems.

## References

[1] Gong Z, Xu K, Meng X, Pei Y, Sun X, Dai Y, et al Effect of optical losses on the transmission performance of a radio-over-fiber distributed antenna system. Chinese Optics Letters. 2013; 11(2):8–11.

[2] Taskhiri MM, Amirhosseini M. Inhomogeneous lens antenna design with fan-beam radiation pattern. Chinese Optics Letters. 2016; 14(12):94–98.

[3] Jia HW, Yang F, Zhong Y, Liu HT. Understanding localized surface plasmon resonance with propagative surface plasmon polaritons in optical nanogap antennas. Photonics Research. 2016; 4(6):293–305. 10.1364/PRJ.4.000293

[4] Ramesh S, Rama T. Millimeter wave dielectric loaded exponentially tapered slot antenna array using substrate integrated waveguide for gigabit wireless communications. Journal of Infrared and Millimeter Waves. 2015; 34(5):513–519.

[5] Xu XY, Wu JY, Nguyen TG, Moein T, Chu ST, Little BE, et al Photonic microwave true time delays for phased array antennas using a 49 GHz FSR integrated optical micro-comb source. Photonics Research. 2018; 6(5):62–68. 10.1364/PRJ.6.000B30

[6] Padmanabhan S, Stephen R, Murthy C, Coupechoux M. Training-based antenna selection for PER minimization: a pomdp approach. IEEE Transactions on Communications. 2015; 63(9):3247–3260. 10.1109/TCOMM.2015.2455504

[7] Gulati N, Dandekar K. Learning state selection for reconfigurable antennas: a multi-armed bandit approach. IEEE Transactions on Antennas and Propagation. 2014; 62(3):1027–1038. 10.1109/TAP.2013.2276414

[8] Zhou M, Cui H, Song L, Jiao B. Transmit-receive antenna pair selection in full duplex systems. IEEE Wireless Communication Letters. 2014; 3(1):34–37.

[9] Yan S, Yang N, Malaney R, Yuan J. Transmit antenna selection with alamouti coding and power allocation in mimo wiretap channels. IEEE Transactions on Wireless Communications. 2014; 13(3):1656–1667. 10.1109/TWC.2013.013014.131248

[10] Wang X, Amin M, Cao X. Analysis and design of optimum sparse array configurations for adaptive beamforming. IEEE Transactions on Signal Processing. 2017; 66(2):340–351. 10.1109/TSP.2017.2760279

[11] Wang X, Wang P, Wang X. Adaptive sparse array reconfiguration based on machine learning algorithms, In: 2018 IEEE International Conference on Acoustics, Speech and Signal Processing. 2018. pp. 1159–1163.

[12] Ares F, Rengarajan S R, Villanueva E, Skochinski E, Morenoet E. Application of genetic algorithms and simulated annealing technique in optimizing the aperture distributions of antenna arrays. In: 1996 IEEE Antennas and Propagation Society International Symposium. 1996. pp. 806–809.

[13] Makki B, Ide A, Svensson T, Eriksson T, Alouini MS. A genetic algorithm-based antenna selection approach for Large-but-Finite MIMO networks. IEEE Transactions on Vehicular Technology. 2017; 66(7):6591–6595. 10.1109/TVT.2016.2646139

[14] Dong J, Xie Y, Jiang Y, Fang L, Shi RH, Xiong D. Particle swarm optimization for joint transmit and receive antenna selection in MIMO systems. In: 2014 IEEE International Conference on Communication Problem-solving. 2014. pp. 237–240.

[15] Joung J. Machine learning-based antenna selection in wireless communications. IEEE Communications Letters. 2016; 20(11):2241–2244. 10.1109/LCOMM.2016.2594776

[16] Bengio Y. Learning deep architectures for AI. 1st ed Boston: Now Publishers Inc; 2009.

[17] Krizhevsky A, Sutskever I, Hinton G. Imagenet classification with deep convolutional neural networks. Communications of the ACM. 2012; 60(2):1097–1105.

[18] Dieleman S, Schrauwen B. Speech recognition with deep recurrent neural networks. In: 2013 International Conference on Acoustics, Speech and Signal Processing. 2013. pp. 6645–6649.

[19] Greff K, Srivastava RK, Koutnik J, Steunebrink BR, Schmidhuber J, LSTM: a search space odyssey. IEEE Transactions on Neural Networks Learning Systems. 2015; 28(10):2222–2232. 10.1109/TNNLS.2016.2582924 27411231

[20] Lecun Y, Bottou L, Bengio Y, Haffner P. Gradient-based learning applied to document recognition. Proceedings of the IEEE. 1998; 86(11):2278–2324. 10.1109/5.726791

[21] Simonyan K, Zisserman A. Very deep convolutional networks for large-scale image recognition; 2014. Preprint. Available from: arXiv:1409.1556v6.

[22] He K, Zhang X, Ren S, Sun J. Deep residual learning for image recognition. In: 2016 IEEE Conference on Computer Vision and Pattern Recognition. 2016. pp. 770–778.

[23] Lecun Y, Jackel L, Bottou L, Cortes C, Denker J, Druckeret H, et al. Comparison of learning algorithms for handwritten digit recognition. In: 1995 International Conference on Artificial Neural Networks. 1995. pp. 53–60.

[24] Matsugu M, Mori K, Mitari Y, Kaneda Y. Subject independent facial expression recognition with robust face detection using a convolutional neural network. Neural Networks. 2003; 16(5):555–559. 10.1016/S0893-6080(03)00115-1 12850007

[25] Zhou Q, Xing J, Chen W, Zhang X, Yang Q. From signal to image: enabling fine-grained gesture recognition with commercial Wi-Fi devices. Sensors. 2018; 18(9):3142 10.3390/s18093142 PMC6165566 30231472

[26] Dieleman S, Schrauwen B. Deep content-based music recommendation. In: 2013 International Conference on Neural Information Processing Systems. 2013. pp. 2643–2651.

[27] Yin W, Kann K, Yu M, Schutze H. Comparative study of CNN and RNN for natural language processing; 2017. Preprint. Available from: arXiv:1702.01923v1.

[28] Lee W, Jo O, Kim M. Application of End-to-End deep learning in wireless communications systems; 2018, Preprint. Available from: arXiv:1808.02394v1.

[29] Tang F, Mao B, Fadlullah ZM, Kato N, Mizutani K. On removing routing protocol from future wireless networks: a real-time deep learning approach for intelligent traffic control. IEEE Wireless Communications. 2018; 25(1):154–160. 10.1109/MWC.2017.1700244

[30] Thing VLL. IEEE 802.11 network anomaly detection and attack classification: a deep learning approach. In: 2017 IEEE Wireless Communications and Networking Conference. IEEE. 2017. pp. 1–6.

[31] Li CQ, Ji JL, Wang QL, Chen X, Li P. Channel state information prediction for 5G wireless communications: a deep learning approach. IEEE Transactions on Network Science and Engineering. 2018.

[32] Ren J, Wang Z. A novel deep learning method for application identification in wireless network. China Communications. 2018; 15(10):73–83. 10.1109/CC.2018.8485470

[33] Cai J, Li Y, Hu Y. Deep convolutional neural network based antenna selection in multiple-input multiple-output system. In: Proceedings of SPIE 10710 Young Scientists Forum. 2017. pp. 1071024.

